# Xanthogranulomatosis of the spleen: a case report

**DOI:** 10.1186/s40792-018-0448-x

**Published:** 2018-04-19

**Authors:** Goshi Fujimoto, Ken Hayashi, Shigetoshi Yamada, Hiroshi Kusanagi, Koichi Honma

**Affiliations:** 1 Department of Gastroenterological Surgery, Kameda Medical Center, Kamogawa, Chiba Japan; 2 Department of Anatomic Pathology, Kameda Medical Center, Kamogawa, Chiba Japan

**Keywords:** Xanthogranulomatosis, Spleen, Hyperlipidemia, Hypertriglyceridemia

## Abstract

**Background:**

Xanthogranulomatous inflammation is recognized as a subtype of cholecystitis; however, it can also occur in other organs. Xanthogranulomatosis of the kidney, bone, ovary, endometrium, vagina, prostate, lymph nodes and pancreas was reported. Herein, we report a case of laparoscopic splenectomy in a patient with xanthogranulomatosis of the spleen that was difficult to diagnose preoperatively.

**Case presentation:**

A 63-year-old man with a past medical history of hyperlipidemia had gradually growing multiple splenic masses, which were revealed on abdominal ultrasonography. Preoperative imaging suggested hamartoma, extramedullary hematopoiesis, or an inflammatory pseudotumor. Although metastatic splenic tumors and malignant lymphoma are atypical, they were considered in the differential diagnosis. Thus, laparoscopic splenectomy was performed. Pathological results confirmed a diagnosis of splenic xanthogranulomatosis. An increase in the postoperative triglyceride levels indicated that hyperlipidemia was the cause of xanthogranulomatosis of the spleen.

**Conclusions:**

Xanthogranulomatosis should be considered in the differential diagnosis of multiple splenic mass lesions in patients with splenomegaly. Additionally, fine-needle aspiration biopsy should be considered for the preoperative diagnosis.

## Background

Although xanthogranulomatous inflammation is recognized as a subtype of cholecystitis, xanthogranulomatosis of the kidney, bone, ovary, endometrium, vagina, prostate, lymph nodes, and pancreas was reported [1, 2]. Here, we report a case of laparoscopic splenectomy in a patient with xanthogranulomatosis of the spleen that was difficult to diagnose preoperatively.

## Case presentation

**Fig. 1:**
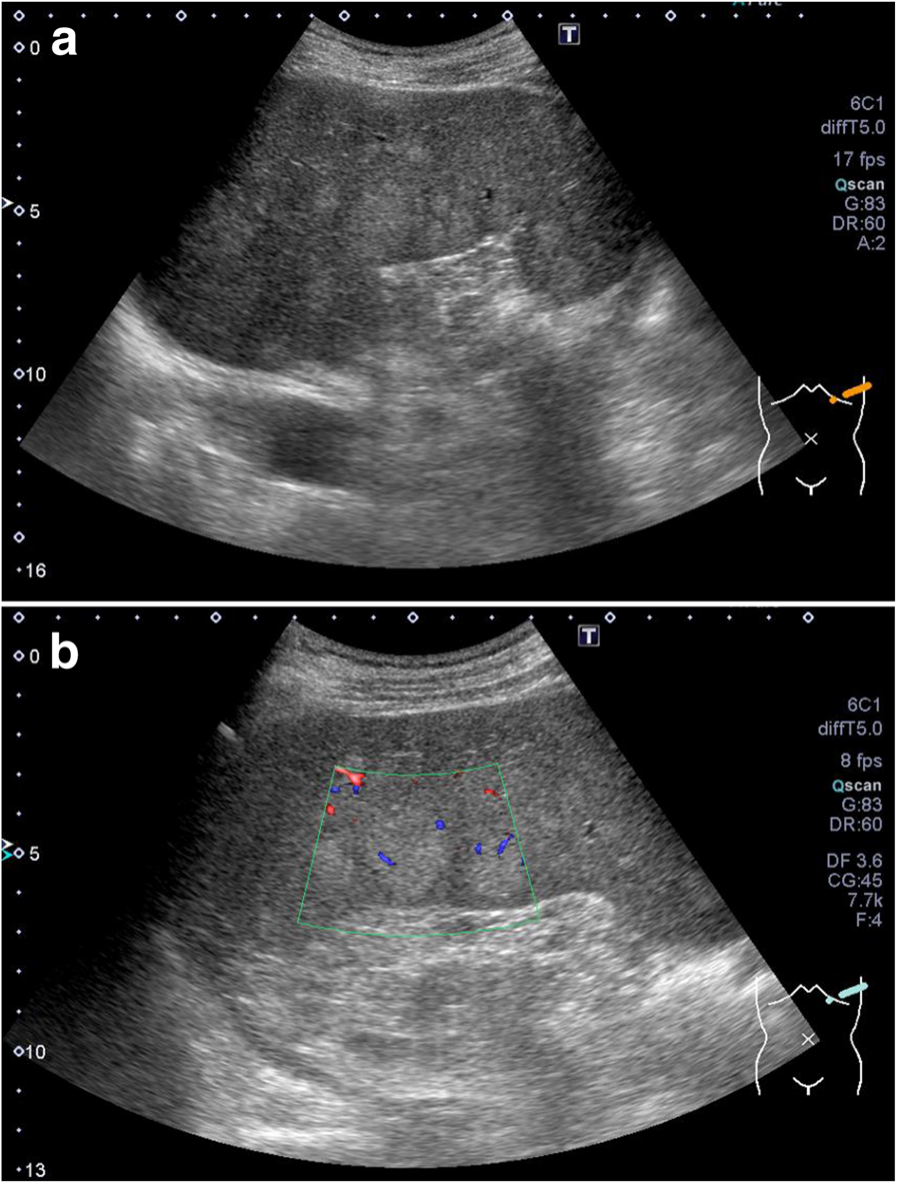
**a** Abdominal ultrasonography findings. Multiple round hyperechoic nodules are seen. **b** Doppler ultrasonography findings. Uneven blood flow is observed in the lesion

**Fig. 2:**
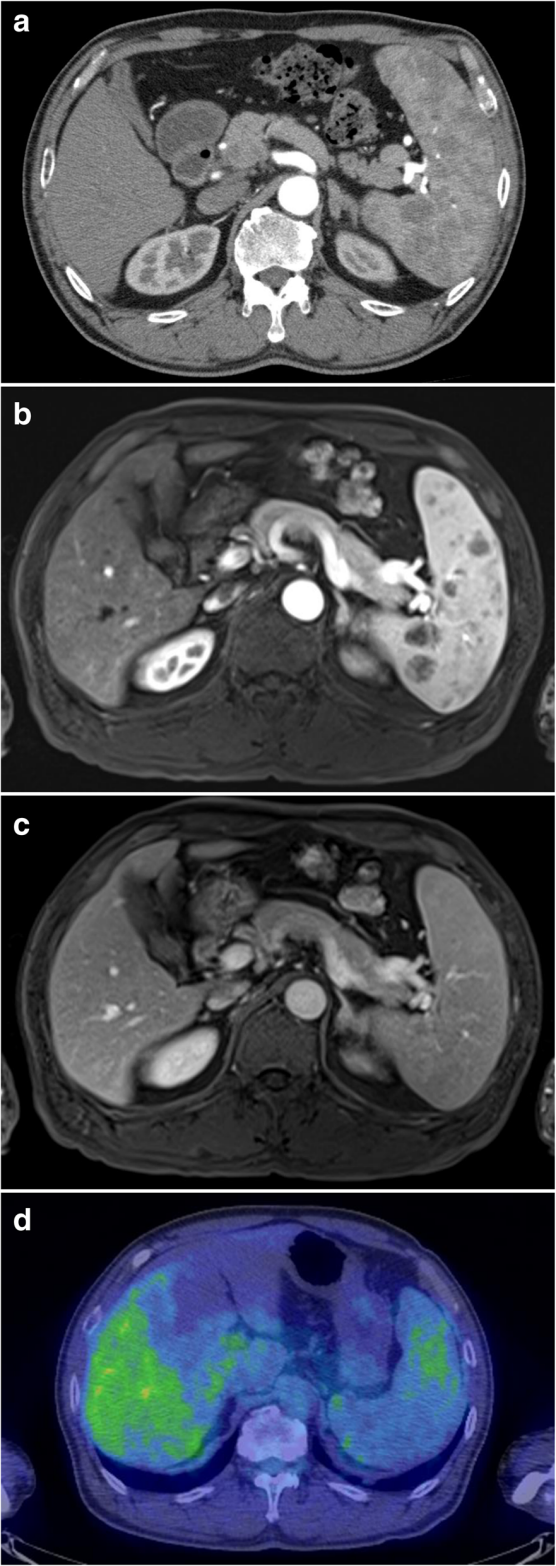
**a** Contrast-enhanced computed tomography findings. Multiple mass lesions of low density are noted in the arterial phase. **b** Findings of dynamic magnetic resonance imaging in the early arterial phase. The mass lesions show low signal intensities. **c** Findings of dynamic magnetic resonance imaging in the equilibrium phase. The mass lesions show signal isointensities. **d** Positron-emission tomography computed tomography findings. Image shows splenomegaly without an abnormal uptake

**Fig. 3:**
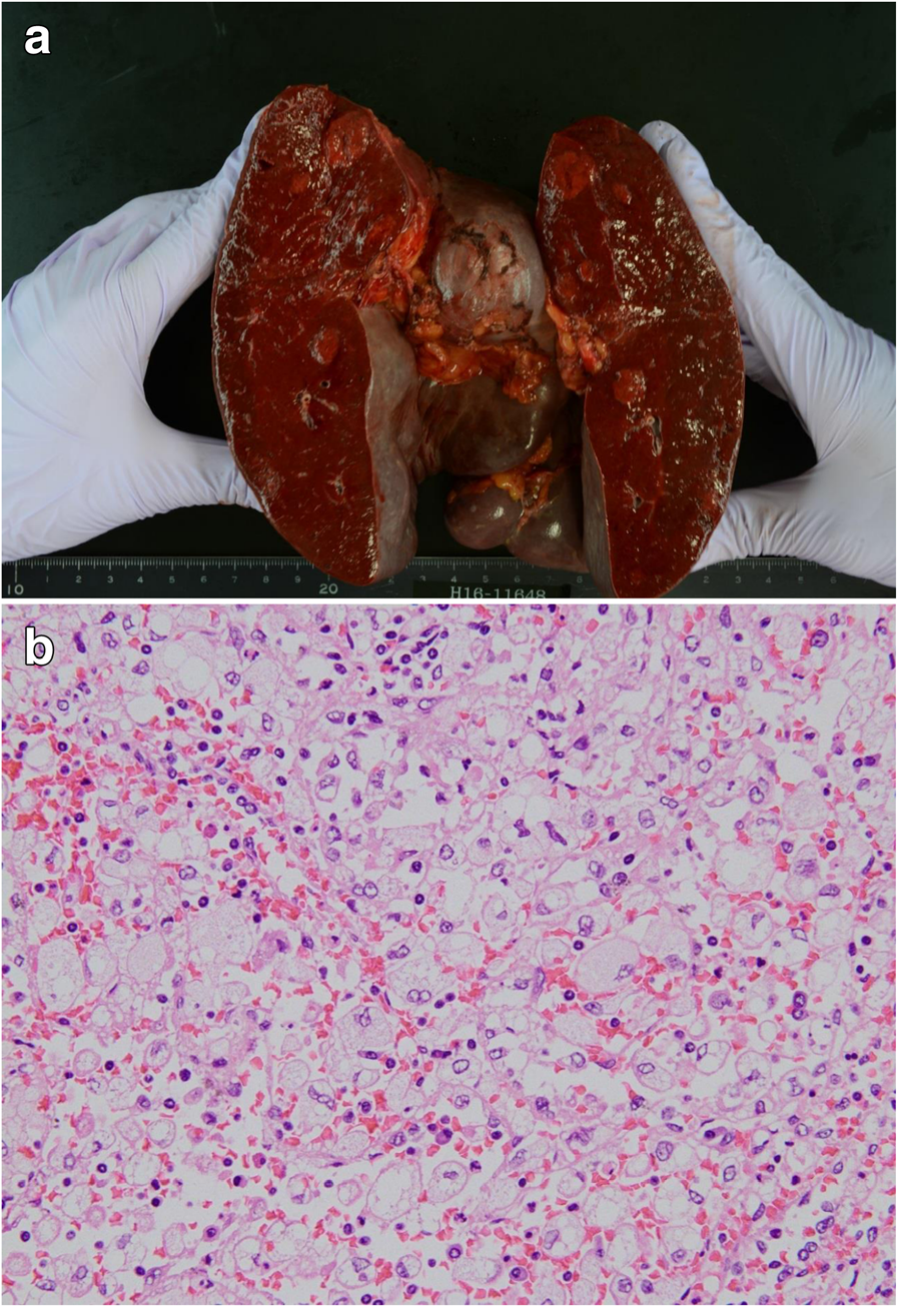
**a** Macroscopic findings. A specimen showing splenomegaly (spleen size, 20 × 14 cm; weight, 940 g) with several elastic soft mass lesions. **b** Microscopic pathology findings. Image shows an extensive collection of polymorphic foamy macrophages

A 63-year-old man with a past medical history of hyperlipidemia had multiple splenic masses, which were revealed on abdominal ultrasonography during a general checkup. His body mass index was 24.9 kg/m^2^. Blood tests revealed an elevated triglyceride (TG) level of 963 mg/dL. On the other hand, total cholesterol (TC) level of 112 mg/dL, low-density lipoprotein cholesterol (LDL-C) level of 15 mg/dL, and high-density lipoprotein cholesterol (HDL-C) level of 16 mg/dL were low. The plasma-soluble interleukin-2 receptor level was normal (Table 1). Bone marrow puncture test was normal. Abdominal ultrasonography (AUS) demonstrated splenomegaly with the spleen measuring 15 × 8 cm. Splenic echotexture was heterogeneous, and multiple round hyperechoic nodules scattered throughout the spleen were noted (Fig. 1a). Uneven blood flow was observed in the lesion on Doppler imaging (Fig. 1b). Contrast-enhanced computed tomography (CT) in the arterial phase revealed low-density multiple masses (Fig. 2a). Magnetic resonance imaging (MRI) confirmed high signal masses on T2-weighted images (Fig. 2b). A low signal intensity in the early arterial phase and an isointensity in the equilibrium phase of dynamic MRI suggested a solid tumor rather than a cyst (Fig. 2c). On positron emission tomography-CT assessment, the splenomegaly without an abnormal uptake was noted (Fig. 2d). There were no remarkable changes on CT and AUS in the following 10 months. We considered hamartoma, extramedullary hematopoiesis, and inflammatory pseudotumor in the differential diagnosis. Additionally, although metastatic splenic tumors and malignant lymphoma are atypical, they were included in the differential diagnosis. Therefore, laparoscopic splenectomy was performed. We encircled the splenic artery in advance and crumped it during surgery to reduce intraoperative blood loss. In addition, a tape was used to encircle and provide traction around the splenic hilum. The total operating time was 321 min, and the total intraoperative blood loss was 75 mL. Although portal vein thrombosis was noted, it improved with anticoagulant therapy. The patient was discharged on day 17 postoperatively. A resected specimen revealed several elastic soft mass lesions (Fig. 3a). Histological sections revealed an extensive collection of polymorphic foamy macrophages (Fig. 3b), which were not S-100 immunoreactive. Epstein-Barr virus early small RNAs (EBER) were negative. Pathology confirmed a diagnosis of splenic xanthogranulomatosis. The following CT a year and a half after surgery showed no other mass lesions. Despite administration of bezafibrate, the hypertriglyceridemia was difficult to control.

**Table 1:** Blood test results of the patient. Blood test results revealed hypertriglyceridemia

Complete blood count		Serum chemistry			
WBC	5400/μL	TP	6.7 g/dL	Na	137 mEq/L
RBC	443 × 10^4^/μL	Alb	4.0 g/dL	K	4.4 mEq/L
Hb	12.6 g/dL	T-Bil	0.6 mg/dL	Cl	102 mEq/L
Ht	35.50%	D-Bil	0.1 mg/dL	Ca	9.1 mg/dL
Plt	11.7 × 10^4^/μL	BUN	18 mg/dL	CRP	0.55 mg/dL
		Cr	0.90 mg/dL	TC	112 mg/dL
Blood coagulation test		LDH	209 IU/L	HDL-C	16 mg/dL
PT (INR)	1.1	CK	70 IU/L	LDL-C	15 mg/dL
PT	82.80%	AST	21 IU/L	TG	963 mg/dL
APTT	39.6 s	ALT	19 IU/L	SIL-2R	281 U/mL

*WBC* white blood cells, *RBC* red blood cells, *Hb* hemoglobin, *Ht* hematocrit, *Plt* platelet, *PT (INR)* prothrombin time (international normalized ratio), *APTT* activated partial thrombin time, *TP* total protein, *Alb* albumin, *T-Bil* total bilirubin, *D-Bil* direct bilirubin, *BUN* blood urea nitrogen, *Cr* creatinine, *LDH* lactate dehydrogenase, *CK* creatinine kinase, *AST* aspartate aminotransferase, *ALT* alanine aminotransferase, *Na* sodium, *K* potassium, *Cl* chlorine, *Ca* calcium, *CRP* C-reactive protein, *TC* total cholesterol, *HDL-C* high-density lipoprotein cholesterol, *LDL-C* low-density lipoprotein cholesterol, *TG* triglyceride, *SIL-2R* soluble interleukin-2 receptor

Xanthogranulomatous inflammation is characterized by the clumping of foamy macrophages and infiltration of inflammatory cells [1, 3]. This lesion is recognized as a subtype of cholecystitis. To our knowledge, this is the first report of a patient with splenic xanthogranulomatosis presenting with multiple masses limited to the spleen. Obstructive conditions and infection can cause xanthogranulomatous changes [1]. Histologically, histiocytosis including Langerhans cell histiocytosis (LCH), hemophagocytic lymphohistiocytosis (HLH), rare Rosai-Dorfmann disease (RDD), Erdheim-Chester disease (ECD), and juvenile xanthogranuloma (JXG) can present with multiple splenic mass lesions. In this case, the cause of xanthogranulomatosis was unclear with respect to the obstructive environment and infection. Macrophages were not S-100 immunoreactive, which suggested that RDD and LCH were unlikely the causes [4, 5]. Secondary HLH, which is usually associated with Epstein-Barr virus (EBV) in Asia, was dismissed following negative test results for EBV receptors [6]. ECD and JXG were dismissed based on physical findings [4, 7]. Other conditions that may cause these splenic lesions include extreme hyperlipidemia and a past history of severe bacteremia. Since the latter did not match the history of the present, hypertriglyceridemia was considered. The spleen is essential for the removal of triglyceride-remnant lipoproteins from the plasma. In addition, enhanced macrophage uptake in an intact spleen contributes to a normal plasma lipid concentration. Thus, splenectomy can result in the development of hypertriglyceridemia [8]. In this patient, postoperative TG level was elevated to 4470 mg/dL, which was the highest value for him. Furthermore, four of his six siblings had hypertriglyceridemia. However, the lipoprotein fraction of the patient and his siblings were not previously evaluated. He has not undergone genetic testing. Low LDL-C level ruled out the possibility of familial hypercholesterolemia [9]. Considering his family history, hereditary disorders causing hypertriglyceridemia, such as familial chylomicronemia, familial hypertriglyceridemia, familial combined hyperlipidemia, and familial dysbetalipoproteinemia, are possible [10–14]. Marked hypertriglyceridemia, low level of TC/TG ratio, low LDL-C level, and low HDL-C level of the patient indicate type I or V hyperlipoproteinemia; therefore, familial chylomicronemia and familial hypertriglyceridemia were suspected. It was reported that hyperlipoproteinemia with hypertriglyceridemia and chylomicronemia causes hepatosplenomegaly [15]. However, the mechanism of multiple mass lesions in this case was unclear. To diagnose this mass lesion preoperatively, fine-needle aspiration biopsy may have been necessary to be considered. Although the risk of bleeding after spleen puncture is reported, the biopsy can be performed safely with a fine needle and an appropriate technique. Among 23 patients who underwent splenectomy soon after fine-needle aspiration biopsy, no intrasplenic hematomas or lesions along the needle path were reportedly observed [16]. If xanthogranulomatosis was revealed from the biopsy, splenectomy might have been avoided.

## Conclusions

Xanthogranulomatosis should be considered in the differential diagnosis in patients with splenomegaly. Additionally, fine-needle aspiration biopsy should be considered for the preoperative diagnosis.

## Abbreviations

AUS: Abdominal ultrasonography
CT: Computed tomography
EBER: Epstein-Barr virus early small RNAs
EBV: Epstein-Barr virus
ECD: Erdheim-Chester disease
HDL-C: High-density lipoprotein cholesterol
HLH: Hemophagocytic lymphohistiocytosis
JXG: Juvenile xanthogranuloma
LCH: Langerhans cell histiocytosis
LDL-C: Low-density lipoprotein cholesterol
MRI: Magnetic resonance imaging
RDD: Rosai-Dorfmann disease
TC: Total cholesterol
TG: Triglyceride
